# Preventive Measures among Healthcare Workers (HCWs) during the COVID-19 Pandemic

**DOI:** 10.3390/ijerph20054434

**Published:** 2023-03-02

**Authors:** Robert Rodríguez-González, Aleksis Galloza, Edgar J. Medina, Valeria Oliver, Natalia I. Rodríguez, Elizabeth Ramos-Colón, Mileily Velázquez-Ferrer, Dayaneira Rivera-Alers, Wanda Vargas, Vanessa Rivera-Amill

**Affiliations:** 1 Public Health Program, Ponce Research Institute, Ponce Health Sciences University, Ponce, PR 00716, USA; 2 School of Medicine, Ponce Health Sciences University, Ponce, PR 00716, USA; 3 Center for Research Resources, RCMI Program, Ponce Research Institute, Ponce Health Sciences University, Ponce, PR 00716, USA

**Keywords:** prevention, COVID-19, SARS-CoV-2, healthcare workers

## Abstract

COVID-19, caused by the severe acute respiratory syndrome coronavirus 2 (SARS-CoV-2), placed health systems worldwide under immense pressure, and healthcare workers (HCWs) were at the front lines. The Puerto Rico Department of Health confirmed the first case of COVID-19 in March 2020. We aimed to assess whether COVID-19 preventive measures implemented by HCWs were effective in a work scenario before vaccine availability. We conducted a descriptive cross-sectional study from July to December 2020 to evaluate the use of personal protective equipment (PPE), hygiene guidelines, and other measures taken by HCWs to prevent the spread of SARS-CoV-2. We collected nasopharyngeal specimens for molecular testing at the beginning of the study and follow-up. We recruited 62 participants aged 30–59 (79% women). Participants recruited from hospitals, clinical laboratories, and private practice included medical technologists (33%), nurses (28%), respiratory therapists (2%), physicians (11%), and others (26%). Among our participants, nurses were at higher risk (*p* < 0.05) of infection. We identified that 87% of participants followed the hygiene recommendation guidelines. In addition, all participants practiced handwashing or disinfection before or after caring for each patient. All participants tested negative for SARS-CoV-2 during the study period. On follow-up, all study participants reported being vaccinated against COVID-19. The implementation of PPE and hygiene measures showed high efficacy as a prevention method against SARS-CoV-2 infection when vaccines and treatment were not widely available in Puerto Rico.

## 1. Introduction

The COVID-19 pandemic had the most significant outbreak in 2020, saturating hospitals, clinics, and other medical facilities worldwide [1]. Healthcare workers (HCWs) are the first line of defense for controlling and preventing severe acute respiratory syndrome coronavirus 2 (SARS-CoV-2) infection [2]. They are constantly at higher risk of becoming infected with SARS-CoV-2 [3]. A meta-analysis from 25 cross-sectional studies determined that the prevalence of COVID-19 among healthcare providers was around 11% [95% CI: 7 to 16%], as detected using RT-PCR tests [4]. Personal shortage of HCWs increases the risk of not having enough professionals to fulfill the infected patients’ needs. Therefore, the burden of COVID-19 among healthcare workers is significant and a cause for global concern [4].

Ensuring the infection prevention of HCWs with COVID-19 is crucial to continue providing the necessary health services. Healthcare workers provide services to improve a population’s quality of life and well-being by carrying out adequate prevention, health promotion, and education [5]. Preventive measures to minimize infection implemented worldwide include using standard personal protective equipment (PPE), which includes surgical masks, gloves, eye protection, surgical gowns, and disinfecting techniques [6]. However, there is a wide variation of compliance by healthcare providers when using personal protective equipment [7].

An international study that includes 2232 healthcare workers from 23 countries different countries confirmed there is variability in PPE use internationally [8]. In Canada, the proportion of healthcare workers that used N-95 (93%), face shields (68%), and gowns (85%) is quite different from that in Italy (79%, 84%, and 69%, respectively) and Spain (71%, 28%, and 68%, respectively) during an interaction with patients that involves contagious aerosol particulate [8]. In the United States, healthcare providers also reported the use of N-95 (71%), face shields (43%), and gowns (79%) when in contact with patients [8].

In Latin American countries, PPE provision is scarce due to pandemic shortages and high demand at different levels [9]. Around 56% of Latin American healthcare workers reported not having access to N-95 masks during patient care [9]. In Peru, 55% of healthcare workers reported receiving PPE for each work shift and 51% received an N-95 mask during their shift [10]. In Latin America, the scarcity of PPE has led healthcare workers to extend the usage time and reuse PPE during interaction with patients [10].

Hygiene guidelines were also implemented to mitigate the spread of COVID-19 [11]. However, SARS-CoV-2 can persist on the skin for at least 8 h at 37 °C and even more if the temperature is lower [12]. Therefore, after being in contact with patients and touching infected surfaces, healthcare providers could have their hands contaminated, leading to the transmission of SARS-CoV-2 and increasing the risk of occupational exposure [12]. The practice of hand hygiene (HH) is a cost-effective measure that could be used to prevent the spread of SARS-CoV-2 infections. However, there is a lack of compliance with the use of hygiene guidelines among healthcare workers [13,14].

In Puerto Rico, as in other countries, the proper use of protective equipment, adherence to preventive measures, and the application of hygiene guidelines among healthcare workers during the COVID-19 pandemic must be evaluated. In addition, the effectiveness of self-care implemented by healthcare providers while caring for patients infected with SARS-CoV-2 still needs to be described. In Puerto Rico, a recent study assessed the PPE used and organizational trust in non-healthcare workers [15]. From this study, 43.5% of non-healthcare workers reported receiving adequate PPE, and 22.4% reported moderate organizational trust [15]. In our study, we aimed to assess whether the COVID-19 prevention measures implemented by healthcare workers were effective in a work setting before the vaccine was available in Puerto Rico.

## 2. Materials and Methods

### 2.1. Ethics Statement

This study was conducted in accordance with the Declaration of Helsinki, and the protocol was approved by the Institutional Review Board of the Ponce Medical School Foundation, Inc. (IRB approval No. 2005037575). All participants signed an informed consent document before sample collection and completion of study questionnaires. 

### 2.2. Study Participants and Sampling

The study involved 62 healthcare professionals aged 21 years or older from the southern region of Puerto Rico. Enrollment of participants was conducted through a convenience sampling process from July 2020 through December 2020. At the beginning of enrollment, a structured questionnaire was applied and once per month, a follow-up screening test was provided to detect positive cases of COVID-19. The participants met the following inclusion criteria: healthcare professionals, ≥21 years old, and currently working in a healthcare scenario. Only those participants who initiated the study process and refused to continue due to personal reasons were excluded. 

### 2.3. Study Design Data Collection Process, and Samples

We conducted quantitative research through a cross-sectional descriptive study to assess behavioral and preventive measures implemented by health professionals on active duty against COVID-19. Healthcare workers from different work scenarios, such as hospitals, clinical labs, reference labs, and clinical offices, were invited to participate. According to each scenario, we collected information on the activities performed by health care professionals and their protective measures implemented against COVID-19.

We used a structured questionnaire that included sociodemographic characteristics, clinical history, exposure to SARS-CoV-2, COVID-19 symptoms, use of protective equipment in work scenarios, preventive and hygiene activities implemented against COVID-19, current health status after interacting COVID-19 positive patients, and once vaccines became available, whether the participants became vaccinated (see questionnaire in Supplementary Materials).

Healthcare professionals were invited to participate in the study using the digital platforms of Ponce Health Sciences University (Web page and E-mail). If participants decided to be in the study, they contacted a research assistant via e-mail or phone. A consent form was completed by the study personnel, followed by a questionnaire and the collection of nasopharyngeal samples. The information was collected using RedCap software (version 12.0.8) and only study personnel and the principal investigator had access to the data.

Nasopharyngeal specimens were tested for the presence of SARS-CoV-2 using an in-house molecular test developed and implemented at Ponce Medical School Foundation, Inc. Immunology Reference Laboratory (a Clinical Laboratory Improvement Amendments (CLIA) and Puerto Rico Department of Health certified laboratory). The protocol was validated and submitted to the Food and Drug Administration under the mechanism of accelerated templates for Laboratories Certified to Perform High Complexity Testing Under CLIA: Emergency Use Authorization. The results of the diagnostic test were provided to study participants.

### 2.4. Statistical Analysis

The data obtained through the research instrument were assessed according to the objectives evaluated. A descriptive analysis was performed for sociodemographic characteristics, including proportions, frequencies, and percentages presented in tables. Histograms, pie charts, and central tendency measures were also used to describe sociodemographic characteristics. The assessment of preventive measures against COVID-19 was initiated by describing the protective equipment used by healthcare professionals. Frequencies, percentages, tables, and a graph bar were used to describe the use of gowns, gloves, surgical masks, N95 masks, and face shields. An association analysis (Fisher test) was performed to obtain odds ratios and assess if protective equipment was associated with the type of exposure (brief interactions or prolonged close contact with COVID-19 patients). Additionally, participants were classified according to their profession and duties. Finally, a comparison of the degree of exposition was assessed by healthcare providers through a Z-test for proportions.

Healthcare providers were asked if they followed hygiene guidelines as recommended and if they used hand sanitizer or soap/water before, during, and after interacting with a COVID-19 patient (frequencies and percentages were calculated). In addition, an assessment of their daily activities after work was performed to determine if they were in contact with people outside their household. Frequencies and percentage of visits to other houses, work/school, healthcare centers, pharmacies, restaurants, gyms, parks, and others were obtained. In addition, an assessment was performed to identify if those participants with a higher degree of educational level were more compromised in following the hygiene guidelines. Data preparation was performed in Excel (version 16.16.27); all analyses were conducted in SPSS (version 28.0.0.0) and STATA (version 13.0).

## 3. Results

### 3.1. General Characteristics of the Study Participants

Table 1 summarizes the general characteristics of the study participants in our study. A total of 62 healthcare workers were eligible and analyzed in the study. Participants were followed during a period of six months (July 2020 through December 2020). The self-descriptive information was obtained through questionnaires. All participants were Hispanic (100%, 62); most of them were females (79%, 49), married (56%, 35), and had a mean age of 44 years (interquartile range [IQR] 20–72). Among the healthcare workers, there were medical technologists (33%, 20), nurses (27%, 17), physicians (16%, 10), and others (24%, 15). In addition, most of the healthcare workers had at least a graduate degree (98%, 61), including an associate’s (8%, 5), bachelor’s (69%, 43), or doctoral degree (21%, 13). Participants were classified according to their work scenarios. Most of the participants were healthcare workers from hospitals (38.7%, N = 24), followed by those who worked at clinical laboratories (21%, N = 13), research laboratories (14.5%, N = 9), private clinics (14.5%, N = 9), pharmacy (4.8%, N = 33) and other (4%, N = 4). Approximately 82% (N = 51) of study participants reported receiving formal training on SARS-CoV-2 infection control and prevention at their current workplace. None of the participants had a positive SARS-CoV-2 RT-PCR test result during the study period. 

### 3.2. Use of Personal Protective Equipment and Prevention Practices

While performing work-related duties, participants used personal protective equipment (PPE), which included surgical masks (95%, N = 59), N95 masks (68%, N = 42), face shields (84%, N = 52), gloves (82%, N = 51), and gowns (84%, N = 52), (Table 2). The proportion of healthcare workers who received training on SARS-CoV-2 infection and practiced the used of PPE was significantly higher (86%, N = 44/51) compared to those who did not receive training and used PPE (63%, N = 7/11) (*p*-value < 0.05).

Most of the healthcare workers (60%, 37) had a low risk of exposition, meaning they had brief interactions with suspected or confirmed COVID-19 patients while wearing approved PPE, while 40% had a medium to high risk of exposition, meaning they had prolonged exposure while using PPE or not using PPE (N = 25). In addition, participants who had prolonged exposure to suspected or confirmed COVID-19 patients had higher odds of using N95-masks (OR = 1.44, 95% CI = 0.37–4.83) and face shields (OR = 4.19, 95% CI = 0.46–39.27). However, personal protective equipment use was not significantly associated with the time of exposition (*p*-value > 0.05). These results indicate that all participants used PPE regardless of the length of exposure. In addition, 87% (N = 54) of the participants confirmed following the recommended hygiene guidelines, and all participants reported practicing handwashing, whether before (94%, 58), or after (95%, 59) seeing a patient (Table 2).

**Figure 1:**
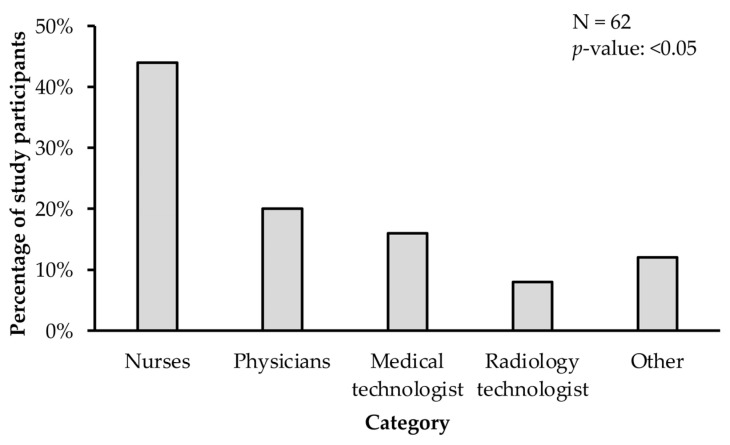
Nurses were among the healthcare workers (HCWs) with a significantly higher risk of exposure to SARS-CoV-2 because of prolonged exposure to a confirmed or suspected case of COVID-19. The proportion of HCWs with medium to high risk of COVID-19 was compared using the proportion Z test. A *p*-value less than 0.05 was considered significant.

Among the healthcare workers that had a prolonged exposure to suspected or confirmed COVID-19 patients (N = 25), nurses (44%, N = 11) were the ones at higher risk (*p*-value < 0.05) compared to physicians (16%, N = 4), medical technologists (16%, N = 4), and radiology technologists (8%, N = 2) (Figure 1).

Once vaccines became available in Puerto Rico in December 2020, we re-contacted study participants to collect information on vaccine administration. All participants reported having received the first dose of the vaccine.

## 4. Discussion

In Puerto Rico, COVID-19 persists, causing outbreaks, and as in other places worldwide, healthcare workers (HCWs) are at a higher risk of infection [16], highlighting the importance of implementing preventive measures. In our study, healthcare workers reported frequent use of personal protective equipment (PPE) such as surgical masks (95%), gowns (83%), face shields (83%), gloves (82%), and N95 masks (68%) when in contact with patients with suspected or confirmed COVID-19. Additionally, all participants reported having implemented handwashing and disinfection practices, including before (93%) and after (95%) seeing a patient. Moreover, for those who had long-time exposure to COVID-19 patients, the odds of N-95 mask used (OR = 1.44, 95% CI = 0.37–4.83) and face shield used (OR = 4.19, 95% CI = 0.46–39.27) were higher, despite PPE not being significantly associated with the length of exposure (*p*-value > 0.05). In addition, most of our participants (87%) confirmed following the recommended hygiene guidelines.

In contrast to our findings, where none of the participants were positive for COVID-19 nor reported disease symptoms and used protective equipment adequately, other studies generally found higher PPE and handwashing non-compliance rates. For example, a study performed in Wuhan, China, described higher rates of poor PPE use practices and handwashing among the younger population [17]. Moreover, Firouzbakht and colleagues, in 2020, noticed higher rates of non-compliance with PPE use and handwashing among healthcare providers from Iran [18]. However, Wang and colleagues, in 2021, in a study from Indonesia, found that the non-compliance rates of PPE use and handwashing among healthcare providers improved following an educational intervention. Therefore, Wang and colleagues stated that misinformation and lack of training are associated with non-compliance among healthcare workers [19].

Razvi and colleagues, in 2020, in a clinical study, also found that healthcare workers with constant patient-facing roles had higher rates of positive COVID-19 antibody tests than HCWs with non-patient-facing roles [20]. Moreover, for healthcare workers with constant-facing roles, such as nurses, the odds of testing positive for COVID-19 antibody tests were double compared to those of HCWs with non-patient-facing roles [20]. Furthermore, a study from Denmark and England confirmed that the percentage of healthcare workers testing positive for SARS-CoV-2 is much higher than that reported by the general population [21].

Healthcare workers well trained on COVID-19 transmission are more likely to maintain preventive measures and follow hygiene guidelines. A study from Assefa and colleagues (2021), which included the assessment of 900 healthcare workers from different countries, Burkina Faso (N = 300), Ethiopia (N = 300), and Nigeria (N = 300), established that nearly all the participants proceeded according to the hygiene guidelines. For example, 89% of the participants from Nigeria received training, compared to 68% from Ethiopia and 62% from Burkina [22]. As a result, 82% of healthcare providers in Nigeria had higher rates of applying preventive measures, compared with 50% in Ethiopia and 39% in Burkina Faso [22]. Similarly, most of our study participants had also received formal training in infection control and prevention at their workplace, which may help explain the high rate of compliance with personal protection measures.

None of our participants tested positive for SARS-CoV-2 during the six-month study period. However, once vaccines became available in Puerto Rico, we re-contacted our study participants and all participants reported being vaccinated. This result suggests that keeping healthcare workers well-trained, promoting the use of protective equipment, and proceeding according to hygiene guidelines, are effective means of protection against SARS-CoV-2 infection when vaccines were not readily available in Puerto Rico. In addition, Rabbani and Al Saigul stated that fear of carrying the virus home to immediate family members might also be why healthcare providers constantly washed their hands in addition to other preventive measures [23].

Our study had some limitations. First, the cross-sectional design limits our ability to make a causal inference. Second, we relied on self-reported information; we need to continue exploring the preventive behavior and personality of the participants. Third, the sample size should be increased in future studies, maintaining the characteristics of a heterogeneous group. Our sample was a heterogeneous group with different professional roles and socioeconomic characteristics.

## 5. Conclusions

Healthcare workers are at constant risk of acting as the first line of defense during pandemics. This study overviews the importance and benefits of keeping a well-trained group of healthcare workers during pandemics. Establishing and promoting personal protective equipment and following the hygiene guidelines as recommended is imperative. The PPE should be used even after considering the time of exposure and distance from the patient. Furthermore, hygiene guidelines should be incorporated not only in the workplace but also in daily activities. As in our study, HCWs followed hygiene guidelines, and PPE was used correctly to reduce the probability of contagion and to get sick. Moreover, the health and performance of healthcare workers were maintained, contributing to the well-being and safety of the patients. Therefore, short training sessions are recommended for healthcare workers to review the use of protective equipment and hygiene guidelines before working with patients during pandemics such as COVID-19.

During a global emergency, the aim is to keep HCWs safe, healthy, and able to work during the emergency response. Our study and others encourage the maintenance of well-trained staff and restate the importance of protective equipment in healthcare facilities. HCWs who are well-trained, follow preventive measures, and adequately use PPE are better prepared to provide services, take care of people, and improve health outcomes. Effective preventive measures, use of PPE, and improvement in containment and control strategies lead to better outcomes among healthcare workers, especially when no treatment or vaccines are available.

## Figures and Tables

**Table 1:** Demographic characteristics of healthcare workers (HCWs) during the COVID-19 pandemic, Puerto Rico, July–December 2020.

Participant Characteristics	(N) 62	(%) 100%
Sex		
Male	13	21.0%
Female	49	79.0%
Age		
21 to 29	12	19.4%
30 to 39	13	21.0%
40 to 49	13	21.0%
50 to 59	15	24.2%
60 to 69	8	12.9%
>70	1	1.6%
Ethnicity		
Hispanic or Latino	62	100%
Race		
White	35	56.5%
Black	16	25.8%
Unknown or Not Reported	11	17.7%
Civil status		
Single	17	27.4%
Married	35	56.5%
Living together (not married)	2	3.2%
Divorced	6	9.7%
Widow	2	3.2%
Education		
Enrolled at university	1	1.6%
Associate degree	5	8.1%
Bachelor’s degree	21	33.9%
Doctoral degree	13	21.0%
Graduated or professional degree	22	35.5%
Profession		
Medical Technologist	20	32.3%
Nurse	17	27.4%
Physician	10	16.1%
Other	15	24.2%
Place of Work		
Hospital	24	38.7%
Clinical laboratory	13	21.0%
Research laboratory	9	14.5%
Private Clinic	9	14.5%
Pharmacy	3	4.8%
Other	4	6.5%
Received training on infection control and prevention	51	82.3%

**Table 2:** Personal protection equipment and practices by healthcare workers and its association with time of exposition.

Parameter	Total N (N = 62)	HCWs with Prolonged Exposure (N = 25)	HCWs with Brief Exposure (N = 37)	aOR (95% CI)	*p*-Value *
Use of Personal Protective Equipment (PPE)					
Surgical mask	59 (95%)	23 (92%)	36 (97%)	0.32 (0.03–3.72)	0.34
N-95 mask	42 (68%)	19 (76%)	23 (62%)	1.44 (0.37–4.83)	0.59
Face shield	52 (84%)	24 (96%)	28 (76%)	4.19 (0.46–39.27)	0.16
Gloves	51 (82%)	21 (84%)	30 (81%)	1.05 (0.26–4.18)	0.94
Gowns	52 (84%)	21 (84%)	31 (84%)	1.35 (0.23–6.78)	0.73
Hand hygiene	62 (100%)	-	-	-	
Before patient care	58 (94%)	22 (88%)	31 (84%)	0.20 (0.02–2.08)	0.18
After patient care	59 (95%)	25 (100%)	34 (92%)	-	-

* Adjusted odds ratios were calculated and adjusted by possible confounder variables such as sex and age; CI, confidence interval.

## Data Availability

Not applicable.

## Supplementary Materials

The following supporting information can be downloaded at: https://www.mdpi.com/article/10.3390/ijerph20054434/s1. Study Questionnaire.
