# Supplementary material for: Preventive Measures among Healthcare Workers (HCWs) during the COVID-19 Pandemic
Source: Int J Environ Res Public Health. 2023 Mar 2;20(5):4434. doi: 10.3390/ijerph20054434 (PMC10002078; doi:10.3390/ijerph20054434)
Supplement: Supplementary file 1 [file ijerph-20-04434-s001.zip › ijerph-2161562-supplementary.pdf]

Study Questionnaire

Please complete the survey below.

Thank you!

Date: ______________________

Participant's Name: ______________________
((Last Name, First Name))

Address (of residence) ______________________

Is the residence a:
- ◯ House
- ◯ Duplex home
- ◯ Apartment building
- ◯ Dorm

How many individuals live in the residence? ______________________

Telephone ______________________

Alternate telephone ______________________

E-mail ______________________

Case Demographics

Date of Birth: ______________________

Age (in years): ______________________

Sex:
- ◯ Male
- ◯ Female
- ◯ Other
- ◯ Unknown

If female, currently pregnant
- ◯ Yes
- ◯ No
- ◯ Unknown

Ethnicity:
- ◯ Hispanic/Latino
- ◯ Non-Hispanic/Latino
- ◯ Unknown

Race (check all that apply):	☐ Black<br>☐ White<br>☐ Asian<br>☐ American Indian<br>☐ Native Hawaiian/Pacific Islander<br>☐ Unknown
Civil State:	◯ Single<br>◯ Married<br>◯ Separated<br>◯ Divorced<br>◯ Widow<br>◯ Cohabitation
Education:	◯ Currently enrolled at university<br>◯ Associate degree<br>◯ Bachelor's degree<br>◯ Graduate or professional degree<br>◯ Doctorate

Healthcare Provider Information

Are you a healthcare provider in Puerto Rico?	◯ Yes<br>◯ No<br>◯ Unknown
If yes, what is your occupation?	◯ Physician<br>◯ Nurse<br>◯ Respiratory therapist<br>◯ Medical technologist<br>◯ Other
If other, specify	______________________________
If yes, what is your job setting?	◯ Hospital<br>◯ Long-term care facility<br>◯ Rehabilitation facility<br>◯ Clinical laboratory<br>◯ Nursing home/assisted living facility<br>◯ Other
If other, specify	______________________________

Exposure Information

	Yes	No
Are you currently sick?	◯	◯

In the 14 days prior to illness onset, did you have any of the following exposures? (Check all that apply.)

	Yes	No
Domestic travel (outside state of normal residence)	◯	◯
Cruise ship or vessel travel as passenger or crew member.	◯	◯
Workplace	◯	◯
Airport/airplane	◯	◯
Adult congregate living facility (nursing, assisted living, or long-term care facility)	◯	◯
School/university/childcare center	◯	◯
Community events/mass gathering	◯	◯
International travel	◯	◯
Contact with a known COVID-19 case (probable or confirmed)	◯	◯
Correctional facility	◯	◯
Animal with confirmed or suspected COVID-19	◯	◯

Domestic travel:

If yes, specify state(s). ______________________

Cruise ship or travel as passenger or crew member.

If yes, specify name of ship: ______________________

International travel:

If yes, specify country(ies). ______________________

Workplace

If yes, is the workplace critical infrastructure (e.g., healthcare setting, grocery store)? ______________________

If you had contact with a known COVID-19 case: What type of contact?

◯ Household contact
◯ Community-associated contact
◯ Healthcare-associated contact

Animal with confirmed or suspected COVID-19:

If yes, specify animal. ______________________

Unknown exposures in the 14 days prior to illness onset: ______________________

Other exposures, specify: ______________________

Use of Personal Protective Equipment

	Yes	No
Gown	◯	◯
Gloves	◯	◯
Surgical mask	◯	◯
N95 mask	◯	◯
Face shield	◯	◯
Hand washing while at work	◯	◯

Re-use gowns ◯ Yes ◯ No

Re-use gloves ◯ Yes ◯ No

Re-use surgical masks ◯ Yes ◯ No

Re-use N95 mask ◯ Yes ◯ No

Re-use Face shield ◯ Yes ◯ No

Gown:

If yes, how many hours per day? ______________________

Gloves:

If yes, how many hours per day? ______________________

Surgical mask:

If yes, how many hours per day? ______________________

N95 mask:

If yes, how many hours per day? ______________________

Face Shield:

If yes, how many hours per day? ______________________

Hand washing while at work:

If yes, how many hours per day?

◯ 0-5
◯ 6-10
◯ >11

If the answer to any of the above questions is no, please explain why:

____

Exposure categories	◯ Are you a healthcare personnel who have had prolonged close contact with suspected/confirmed COVID-19 patients while NOT wearing personal protective equipment (PPE)<br>◯ Are you a healthcare personnel who had prolonged close contact with suspected/confirmed COVID-19 patients while wearing approved PPE<br>◯ Are you a healthcare personnel who had brief interactions with suspected/confirmed COVID-19 patients while wearing approved PPE

Compliance with infection prevention and control measures

Did you receive training related to infection control and prevention?	◯ Yes<br>◯ No
If yes, when did you receive training? (MM/DD/YYYY)	______________________
How many hours of training did you receive?	◯ Less than 2 hours<br>◯ More than 2 hours
Do you follow the hygiene guidelines as recommended?	◯ Yes, as recommended<br>◯ Most of the time<br>◯ Occasionally<br>◯ Rarely
Do you use alcohol-based hand sanitizer or soap and water before touching each patient?	◯ Yes, as recommended<br>◯ Most of the time<br>◯ Occasionally<br>◯ Rarely
Do you use alcohol-based hand sanitizer or soap and water after touching each patient, their fluids, or their surroundings?	◯ Yes, as recommended<br>◯ Most of the time<br>◯ Occasionally<br>◯ Rarely
Is there enough personal protective equipment in your workplace?	◯ Yes<br>◯ No<br>◯ Unknown
Handwashing while in the workplace	◯ Yes<br>◯ No

Clinical Course, Symptoms, Past Medical History, and Social History

Have you experienced any symptoms in the past 2 weeks?	◯ Yes, symptomatic<br>◯ No symptoms
Onset date of symptoms (MM/DD/YYYY):	______________________
If symptomatic, did your symptoms resolve?	◯ Yes<br>◯ No, still symptomatic<br>◯ Symptoms resolved, unknown date<br>◯ Unknown if symptoms resolved

If yes,

Date of symptom resolution (MM/DD/YYYY): ________________

	Yes	No	Unknown	N/A
Did you develop pneumonia?	◯	◯	◯	◯
Did you have acute respiratory distress syndrome?	◯	◯	◯	◯
Did you have an abnormal chest X-ray?	◯	◯	◯	◯
Did you have another diagnosis/ etiology for the illness?	◯	◯	◯	◯
Did you have an abnormal EKG?	◯	◯	◯	◯
Did you receive extracorporeal membrane oxygenation?	◯	◯	◯	◯
Did you receive mechanical ventilation (MV)/intubation?	◯	◯	◯	◯

If yes,

Total days with MV: ________________

If symptomatic, which of the following did you experience during the illness?

	Yes	No	Unknown
Fever >100.4F (38C)	◯	◯	◯
Subjective fever (felt feverish)	◯	◯	◯
Chills	◯	◯	◯
Rigors	◯	◯	◯
Muscle aches (myalgia)	◯	◯	◯
Runny nose (rhinorrhea)	◯	◯	◯
Sore throat	◯	◯	◯
New olfactory and taste disorders	◯	◯	◯
Headache	◯	◯	◯
Fatigue	◯	◯	◯
Joint pain	◯	◯	◯
Nasal bleeding	◯	◯	◯
Conjunctivitis	◯	◯	◯
Cough (new onset or worsening of chronic cough)	◯	◯	◯
Difficulty breathing	◯	◯	◯
Chest pain	◯	◯	◯

Nausea or vomiting	○	○	○
Abdominal pain	○	○	○
Diarrhea (>3 loose stools/24hr period)	○	○	○
Rash	○	○	○
Were you hospitalized?	○	○	○

Other symptoms? Specify: ______________________

If hospitalized:

Date of hospitalization (MM, DD, YYYY) ______________________

If hospitalized,

Specify the reason: ______________________

Do you have any of the following underlying medical condition and/or risk behaviors?

	Yes	No	Unknown
Diabetes	○	○	○
Hypertension	○	○	○
Severe obesity (BMI >40)	○	○	○
Cardiovascular disease	○	○	○
Chronic Renal disease	○	○	○
Chronic Liver disease	○	○	○
Arthritis	○	○	○
Chronic Lung disease (asthma/emphysema/COPD)	○	○	○
Immunosuppresive condition	○	○	○
Autoimmune condition	○	○	○
Current smoker	○	○	○
Former smoker	○	○	○
Second-hand smoker	○	○	○
Alcohol	○	○	○
Substance abuse or misuse	○	○	○
Disability (neurologic, physical, vision or hearing impairment	○	○	○
Psychological/psychiatric condition	○	○	○
Other chronic diseases	○	○	○
Other underlying condition or risk behavior	○	○	○

Diabetes:

If yes, specify type: ______________________

Disability:

If yes, specify: ______________________

Other Chronic disease:

If yes, which other chronic disease? ______________________

Other Underlying condition or risk behavior

If yes, specify: ______________________

Sample Collection

Choose a day for sample collection:
(Please choose a day Monday-Tuesday or Thursday-Friday) ______________________

Choose a time:
- ○ 8:00am-12:00pm
- ○ 1:00pm-2:30pm

COVID-19 Vaccination

	Yes	No
Are you vaccinated against COVID-19?	○	○

Which vaccine did you receive?
- ○ Pfizer
- ○ Moderna
- ○ Johnson and Johnson

Date of First Dose ______________________

Date of Second Dose ______________________
